# 

**Published:** 1849-01

**Authors:** 


*Convention of Delegates from Western Schools.*—The Editor of the Ohio Med. and Surg. Jour., in a late number, suggested a convention of delegates from Western schools, to meet at Cincinnati, Ohio, in order to consult upon matters relating to medical education, with a view of uniformity in fees, duration of lecture terms, and other matters. The object appears to us highly important, and the proposition is advocated by the Western Lancet, and the Western Med. and Surg. Jour. Although not authorized to speak on behalf of the Buffalo Medical School, we presume the proposition would be acceptable to the Faculty of that Institution, should all the other schools unite upon it. It strikes us that, to save the trouble of a convention at Cincinnati, delegates from all of the schools attending the meeting of the American Medical Association in May next, might hold their convention at the same time and place. An additional reason for this is, that the action of the convention might be influenced by the action of Eastern schools, and the proceedings of the Association. The Buffalo College has already gone as far as any other medical institution of the Union, in adopting the recommendations of the American Association, which is a pretty good guaranty that she will not be backward in her concurrence with other

Western schools, in any action which promises to forward the objects of medical education, or the best interests of the Profession.

---

*Delegates to the American Medical Association.*—The committee of arrangements request that societies, and other institutions authorized to appoint delegates, send correct lists of those chosen to attend the next annual meeting, to *Dr. Henry J. Bowditch*, Boston, on or before April 1, 1848.

---

*New Year—Eheu! fugaces, Postume, Postume, Labuntur Anni.*—Another annual stadium of the journey of life is completed! Another new-year's day, like the mile stone, reminds us that we are hastening onward, with rail road speed, to that goal—the common destiny of man—when years will cease to be numbered. It is a season signalized by joyous hilarity, but, to the reflecting mind, it is also a time for sober meditations. Our readers may, perhaps, congratulate themselves, that we have no space for editorial moralizing. The compositor limits us to a few lines—just to express to our patrons our cordial greetings, and fervent wishes that the commencement of a new year may prove to each and all, an occasion for happy retrospections, and bright anticipations.

---

*To Correspondents.*—We occasionally receive for insertion, in this Journal, reports in which reflections upon treatment pursued by other physicians in the case, are implied. To obviate the necessity, in future, of rejecting such communications, we would state, that this will always be with us a sufficient ground for declining their publication. Whenever we receive such an article, we suspect that improper motives have actuated the writer; and, apart from our indisposition to lend our pages for any sinister objects, the facts purported to be set forth, are, as we think, invalidated by the occasion for such a suspicion.

Dr. Hard's communication has been received, and will appear in our next number.

We would acknowledge the receipt of Dr. D's letter, to which we will respond.

Books received, and not yet noticed, will receive attention as early as convenience will permit.

